# Longitudinal dynamics of the nasopharyngeal microbiome in response to SARS-CoV-2 Omicron variant and HIV infection in Kenyan women and their children

**DOI:** 10.1128/msystems.01568-24

**Published:** 2025-04-22

**Authors:** Ayla Žuštra, Victoria R. Leonard, LaRinda A. Holland, James C. Hu, Tianchen Mu, Steven C. Holland, Lily I. Wu, Emily R. Begnel, Ednah Ojee, Bhavna H. Chohan, Barbra A. Richardson, John Kinuthia, Dalton Wamalwa, Jennifer Slyker, Dara A. Lehman, Soren Gantt, Efrem S. Lim

**Affiliations:** 1 School of Life Sciences, Arizona State University, Tempe, Arizona, USA; 2 Center for Fundamental and Applied Microbiomics, Biodesign Institute, Arizona State University, Tempe, Arizona, USA; 3 Department of Global Health, University of Washington 7284, Seattle, Washington, USA; 4 Department of Paediatrics and Child Health, University of Nairobi 107854, Nairobi, Kenya; 5 Kenya Medical Research Institute 118982, Nairobi, Kenya; 6 Department of Biostatistics, University of Washington 7284, Seattle, Washington, USA; 7 Department of Research and Programs, Kenyatta National Hospital 285569, Nairobi, Kenya; 8 Department of Epidemiology, University of Washington 7284, Seattle, Washington, USA; 9 Division of Human Biology, Fred Hutchinson Cancer Research Center 7286 , Seattle, Washington, USA; 10 Département de Microbiologie, Infectiologie et Immunologie, Université de Montréal, Centre de Recherche du CHU St-Justine, Montréal, Québec, Canada; 11 National Centre for Infectious Diseases, Singapore, Singapore; Northern Arizona University, Flagstaff, Arizona, USA

**Keywords:** nasopharyngeal microbiome, SARS-CoV-2, COVID-19, human immunodeficiency virus, mother-infant, genomic epidemiology, longitudinal

## Abstract

The nasopharynx and its microbiota are implicated in respiratory health and disease. The interplay between viral infection and the nasopharyngeal microbiome is an area of increased interest. The impact of severe acute respiratory syndrome coronavirus 2 (SARS-CoV-2), the etiological agent of the coronavirus disease 2019 pandemic, on the nasopharyngeal microbiome among individuals living with HIV is not fully characterized. Here, we describe the nasopharyngeal microbiome before, during, and after SARS-CoV-2 infection in a longitudinal cohort of Kenyan women (21 living with HIV and 14 HIV-uninfected) and their children (18 HIV-exposed, uninfected and 7 HIV-unexposed, uninfected) between September 2021 and March 2022. We show using genomic epidemiology that mother and child dyads were infected with the same strain of the SARS-CoV-2 Omicron variant that spread rapidly across Kenya. We used metagenomic sequencing to characterize the nasopharyngeal microbiome of 20 women and children infected with SARS-CoV-2, six children negative for SARS-CoV-2 but experiencing respiratory symptoms, and 34 timepoint-matched SARS-CoV-2-negative mothers and children. Since individuals were sampled longitudinally before and after SARS-CoV-2 infection, we could characterize the short- (within a week of infection) and longer- (average of 38 days post-infection) term impact of SARS-CoV-2 infection on the nasopharyngeal microbiome. We found that mothers and children had significantly different microbiome composition and bacterial load (*P*-values < 0.0001). In both mothers and children, the nasopharyngeal microbiome did not differ before and after SARS-CoV-2 infection, regardless of HIV exposure status. Our results indicate that the nasopharyngeal microbiome is resilient to SARS-CoV-2 infection and was not significantly modified by HIV.

**IMPORTANCE:**

The nasopharyngeal microbiome plays an important role in human health. The degree of impact that severe acute respiratory syndrome coronavirus 2 (SARS-CoV-2) infection has on the nasopharyngeal microbiome varies among studies and may be influenced by diverse SARS-CoV-2 variants and variations in the microbiome between individuals. Our results show that the nasopharyngeal microbiome was not altered substantially by SARS-CoV-2 infection nor by HIV infection in mothers or HIV exposure in children. Our findings highlight the resilience of the nasopharyngeal microbiome after SARS-CoV-2 infection. These findings advance our understanding of the nasopharyngeal microbiome and its interactions with viral infections.

## INTRODUCTION

The nasopharyngeal (NP) microbiome plays an important role in overall respiratory health and mucosal homeostasis (1). Interactions and perturbations in the microbiome have been associated with increased susceptibility to respiratory infections, such as influenza (2). The mature adult nasopharynx is colonized by keystone commensal microorganisms such as *Dolosigranulum* spp., *Moraxella* spp., and *Corynebacterium* spp., as well as traditionally pathogenic microorganisms, namely *Haemophilus* spp., *Staphylococcus aureus*, and *Streptococcus pneumoniae*, of which commensal species of the *Staphylococcus* and *Streptococcus* genera also colonize the mature nasopharynx (3–5). The nasopharyngeal microbiome in healthy infants is different from the mature adult microbiome, with conflicting reports on what age the infant nasopharyngeal microbiome begins to resemble the adult microbiome (3). The infant nasopharyngeal microbiome is highly dynamic within the first year of life (4), and is influenced by both host and environmental factors such as antibiotics, birth mode, feeding type, seasonality, and vaccination (5).

Dysbiosis in both the infant and adult nasopharyngeal microbiomes can result in increased susceptibility to severe lower respiratory tract infections (6). Specifically, individuals infected with metapneumovirus had reduced aerobic bacteria and increased pathogenic bacteria in their nasopharynx (7). Colonization of the early infant nasopharyngeal microbiome by *Streptococcus pneumoniae*, *Haemophilus influenzae*, and *Moraxella catarrhalis* is shown to increase the risk of pneumonia and bronchiolitis, independent of childhood asthma diagnosis (8). Additionally, respiratory syncytial virus or human rhinovirus infection in infants may lead to perturbations in bacterial genera significantly associated with childhood asthma development, such as *Streptococcus*, *Moraxella*, and *Haemophilus* (9). Similarly, in both infants and adults, it has been shown that certain microbiome community states in the nasopharynx are associated with influenza infection susceptibility, though the intricacies of this interaction have yet to be elucidated (2). Overall, there is complex interplay between viral infection, nasopharyngeal microbial composition, and subsequent susceptibility to respiratory infections (6).

Severe acute respiratory syndrome coronavirus 2 (SARS-CoV-2) is the etiological agent of the coronavirus disease 2019 (COVID-19) pandemic and since its emergence in 2019 has evolved multiple variants of concern (VOCs). Delta and Omicron were designated VOCs in May and November 2021, respectively (10). The emergence of Omicron occurred both quickly and abundantly (11, 12), and was first observed in Kenya in November 2021, where it replaced Delta as the primary VOC in the country by mid-December (13). There are functional differences when comparing Delta and Omicron infections. Compared to infections with Omicron variants, those infected with Delta had more severe symptoms for a longer period of time, as well as an increased risk of hospitalization (14). Individuals who were infected with Delta were also more likely to experience long COVID (15), and when infected, individuals were told to self-quarantine at home, which resulted in increased transmission within the home (16). Thus, varying levels of disease severity due to different variants of SARS-CoV-2 infection may have distinct impacts on other aspects of human health, including the microbiome.

There is not yet a clear association between the microbiome and SARS-CoV-2. Studies have shown SARS-CoV-2 infection can cause an overall disruption of the gut microbiota marked by a decrease in commensal bacteria and an increase in pathogenic bacteria (17). These disruptions in the gut microbiome can lead to subsequent infections or deterioration of the gut lining, exacerbating the risk for secondary infections (18). The relationship between SARS-CoV-2 infection and the nasopharyngeal microbiome is less clear. Some studies have reported a decrease in specific bacterial species or a decrease in overall alpha diversity (19, 20), while others have reported no significant differences in the nasopharyngeal microbiota (21, 22). Interestingly, studies in which patients had severe COVID-19 symptoms or were hospitalized reported observable differences in specific nasopharyngeal bacterial taxa compared to mild/non-symptomatic cases (23–25). However, studies documenting differences in the nasopharyngeal microbiome were not longitudinal (19, 20), therefore posing challenges in elucidating whether the observed dysbiosis in the microbiome was present pre-infection or a result of COVID-19 infection.

HIV infection and the use of antiretroviral therapies have also been implicated in dysbiosis of the microbiome, with a decrease in bacterial richness and diversity in the gut (26). Given the importance of the nasopharyngeal microbiome in HIV (27, 28), and the impact of SARS-CoV-2 on various microbiomes, further investigation on the impact of SARS-CoV-2 on the nasopharyngeal microbiome is needed. In this study, we aim to characterize the maternal and pediatric nasopharyngeal microbiomes of women living with HIV, HIV-uninfected women, and their children to determine if SARS-CoV-2 and/or HIV have an impact on the nasopharyngeal microbiome during the peak of SARS-CoV-2 Omicron transmission in Kenya.

## RESULTS

### Study design and SARS-CoV-2 infection in a Kenyan mother-child cohort from September 2021 to March 2022

**Fig 1:**
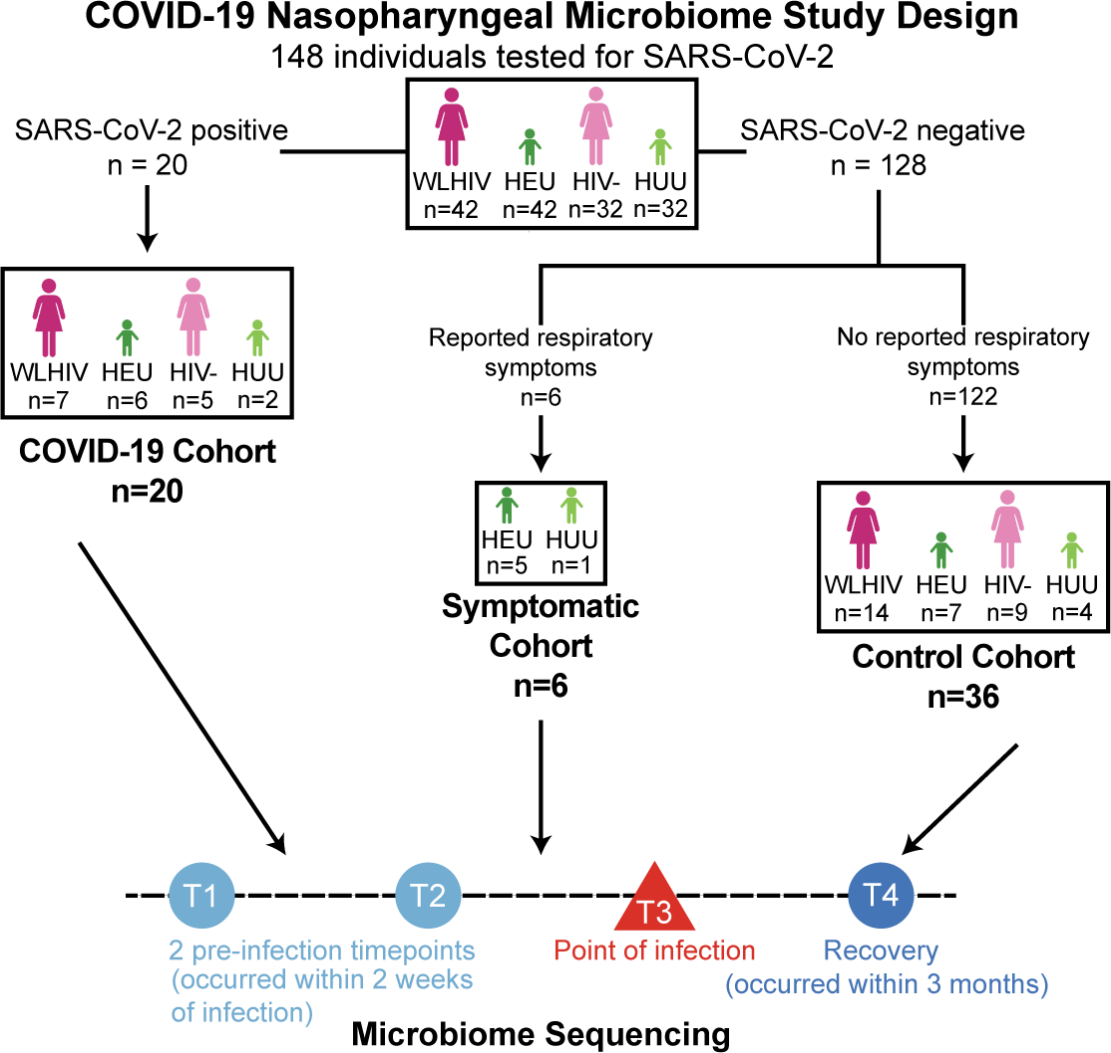
COVID-19 nasopharyngeal microbiome study design. Overview of study design.

We performed a study of Kenyan women and their children enrolled in a prospective cohort in Nairobi, Kenya, who agreed to SARS-CoV-2 testing, as previously described (29). Seventy-four mother-child pairs were included in the parent study: 42 women living with HIV (WLHIV), 32 HIV-uninfected women, and their 74 children—42 HIV-exposed uninfected (HEU) children, 32 HIV-unexposed uninfected (HUU) children (Fig. 1; Fig. S1). WLHIV were given cotrimoxazole to prevent opportunistic infections during high-risk periods, such as pregnancy and breastfeeding, per Kenya Ministry of Health recommendations. Additionally, HEU children received cotrimoxazole as a prophylactic between 4 and 6 weeks old until cessation of breastfeeding and confirmed HIV-uninfected, at approximately 18 months, though this age may vary (30). The children in the study cohort were aged 17–25 months old, of which only three (4%) were still receiving cotrimoxazole prophylaxis since most had stopped breastfeeding. The median age of the women in the cohort was 32, and the median age of the children in the cohort was 21 months (Table S1).

For the nested study, nasopharyngeal swabs were collected for weekly COVID-19 testing (via TaqPath 2.0 qRT-PCR assay) from September 2021 to March 2022, resulting in a total of 1,262 unique swabs from 148 individuals available for this study (average of 8.5 swabs per person over 7 months). Among these, 20 individuals (13.5%) had a single swab test positive for SARS-CoV-2 (1.6% of all samples). The 20 individuals testing positive for SARS-CoV-2 included seven WLHIV, five HIV-uninfected women, six HEU children, and two HUU children; there were four mother-child dyads in which both mother and child were SARS-CoV-2-positive (Fig. 1; Table S1). One individual tested positive in October 2021, 3 in late November 2021, 15 in mid-December 2021, and 1 in January 2022. Six children negative for SARS-CoV-2 with reported respiratory symptoms at the peak of SARS-CoV-2 cases in Kenya at the end of 2021 were included as a symptomatic group. Through hybrid capture next-generation sequencing, we determined that of the six symptomatic children, one child was infected with WU polyomavirus, and three children were infected with different strains of human adenovirus, two of which were co-infected with different strains, and we were unable to determine an etiology for the other two children (Table S2). Additionally, 34 individuals who tested negative for SARS-CoV-2 infection at all time points were included as a comparator group, matched according to HIV status and calendar time points: 14 WLHIV, 32 HIV-uninfected women, 7 HEU children, and 4 HUU children (Fig. S2). Uniquely, unlike earlier studies of SARS-CoV-2 infection, women and children were prospectively sampled longitudinally during the COVID-19 pandemic, allowing us to document changes in the nasopharyngeal microbiome before, during, and after SARS-CoV-2 infection. The median of the next-generation sequencing reads was 7,058,076 (interquartile range [IQR] = 2,408,619–26,567,952).

**Fig 2:**
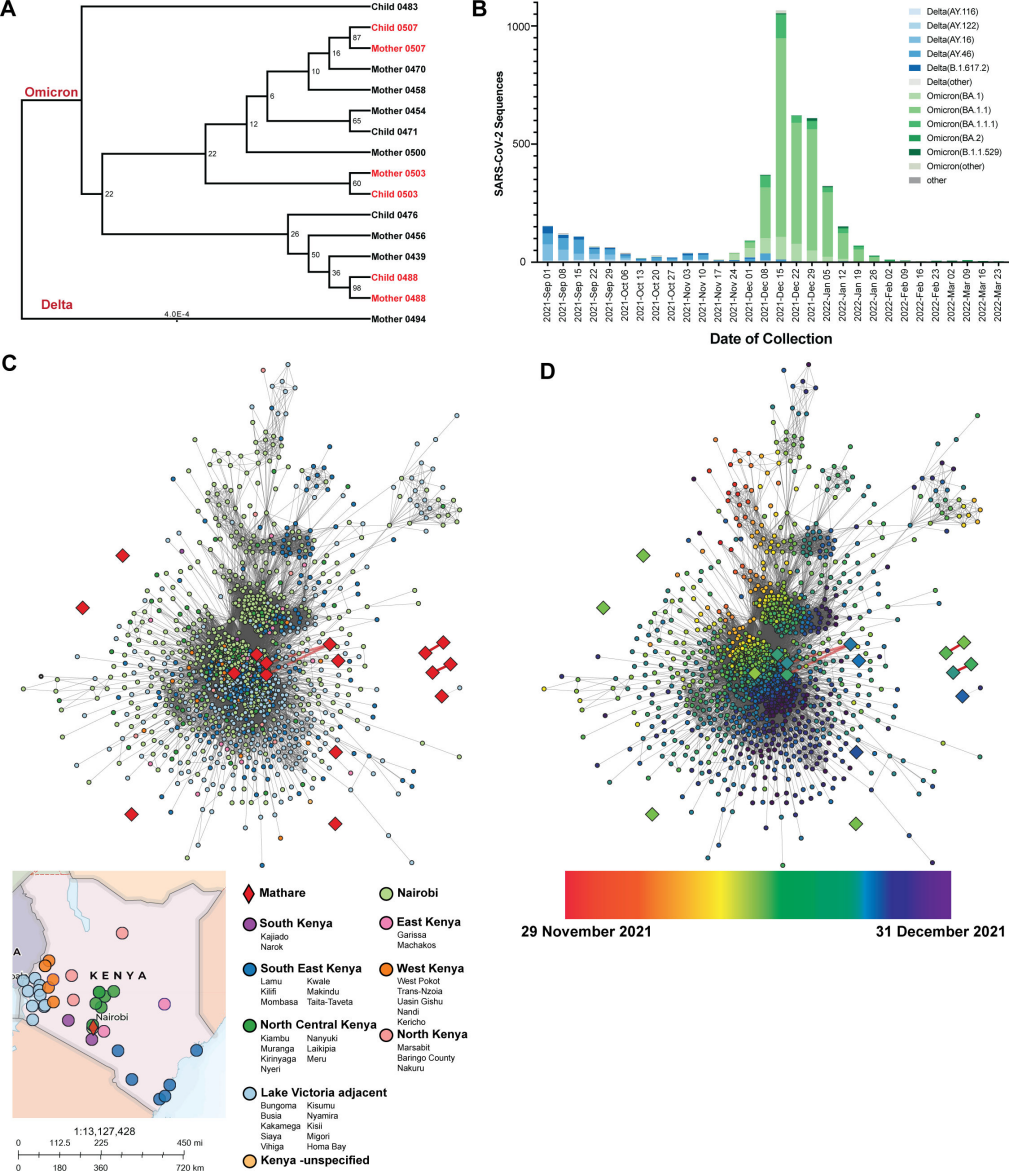
SARS-CoV-2 variant infections among study participants in Mathare and relationship with transmission patterns in Kenya. (**A**) Maximum-likelihood phylogenetic tree of positive SARS-CoV-2 individuals with successful lineage determinations. Mother-child dyads are highlighted in red. Delta is manually designated as outgroup. (**B**) Sublineage frequencies of circulating SARS-CoV-2 genomes shown by weekly collection date in Kenya. (**C**) Kenya country map showing geographic areas where sequences were reported. Thirty-six different cities were categorized into the following groups: North Kenya, North Central Kenya, East Kenya, West Kenya, Lake Victoria adjacent, South Kenya, and Southeast Kenya. Nairobi was considered its own group due to sequence abundance. Mathare was considered an independent group. Sequences in which the geographic metadata was not specified beyond Kenya were grouped independently as well. Visualization of all submitted SARS-CoV-2 genomes in Kenya of the generated transmission network. Nodes are colored by geographic area, provided by GISAID reported metadata, and grouped into larger clusters for visualization purposes. (**D**) Visualization of all submitted SARS-CoV-2 genomes in Kenya of the generated transmission network. Nodes are colored continuously by collection date, provided by GISAID reported metadata, beginning 29 November and ending 31 December 2021.

We first sought to determine which SARS-CoV-2 variants caused infection in the cohort. Through whole-genome sequencing, we identified 15 individuals infected with the Omicron variant, 1 individual infected with the Delta variant, and 4 individuals for whom there was insufficient genome coverage to successfully determine the variant. We constructed a phylogeny using the 16 SARS-CoV-2 sequences (Fig. 2A). Sequences from three mother-child dyads suggest that the mothers and children who were SARS-CoV-2-positive at the same time were infected with the same specific strain of SARS-CoV-2. Conversely, this also suggests the other 14 individuals who only had a mother or child positive for SARS-CoV-2 had discordant transmission.

To compare results of specific SARS-CoV-2 variant transmission in our Nairobi-based cohort to sequences circulating more widely in Kenya, we performed a sequence network transmission analysis using the sequences from our cohort and 3,016 SARS-CoV-2 sequences from 36 different Kenyan cities and counties submitted to GISAID during the peak of COVID-19 cases both in Kenya and in the study cohort (November 2021–December 2021; Fig. 2B). November and December 2021 were when most of the study cohort was positive for COVID-19 as well, showing the epidemiology seen in the cohort is comparable to national Kenyan statistics. To examine the introduction of SARS-CoV-2 throughout Kenya, we analyzed the collection dates and locations of samples from GISAID within sublineages. There is no clear clustering by geographic area (Fig. 2C), but when collection date information is added, we observe samples from the Nairobi region coinciding with earlier time points (Fig. 2D). This would support a scenario where the Nairobi region served as the primary point of SARS-CoV-2 ingress and was spread to the surrounding regions. These data show the cohort samples were consistent with contemporaneous variants circulating in Kenya during the study period.

### The nasopharyngeal microbiome community states in women and children

**Fig 3:**
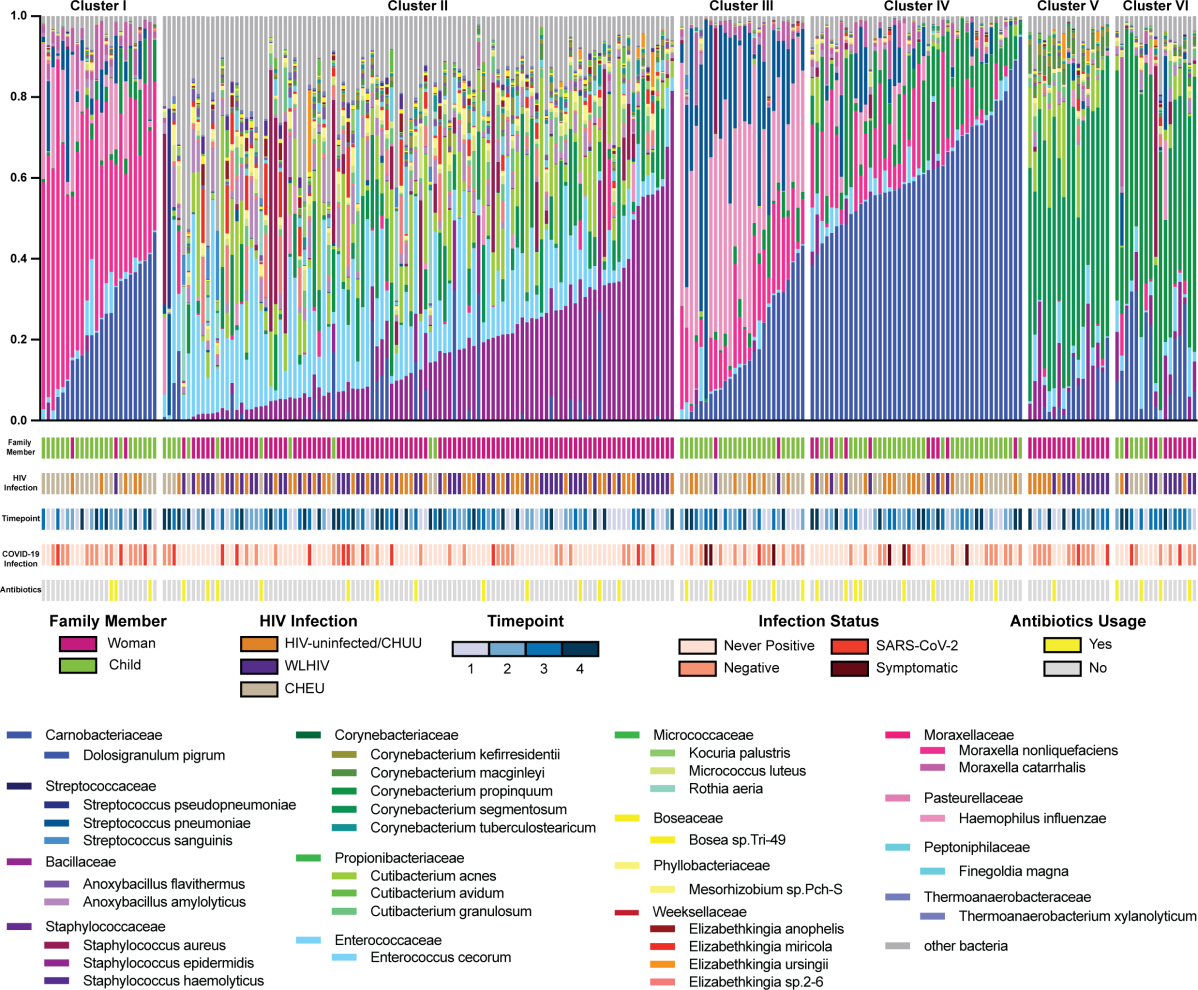
Nasopharyngeal microbiome community states. Relative abundance of bacteria at species level, clustered using k-means. All statistical differences between clusters are due to differences in woman and child samples. Clusters I and II, II and III, and II and IV are statistically different by *P*-values of 0.01. Clusters I and V, I and VI, II and VI, III and V, and VI, and IV and are statistically different by *P*-values of 0.05.

To determine the community state profiles present in the mothers and children, we clustered the nasopharyngeal bacterial microbiome abundance at the species level using the k-means method, resulting in six clusters from every time point from every participant (Fig. 3). Cluster II was the largest cluster with 106 samples and *Staphylococcus epidermidis* (18% relative abundance) and *Enterococcus cecorum* (16% relative abundance) were the most abundant bacterial species. *Dolosigranulum pigrum* (23%, 17%, and 62% relative abundance respectively) was the most abundant bacterial species for clusters I, III, and IV, respectively. *Moraxella nonliquefaciens* was most abundant in clusters I and III (45% and 12% relative abundance respectively), and cluster III additionally had 33% *Haemophilus influenzae* relative abundance and 24% *Streptococcus pneumoniae* relative abundance. Cluster V was predominantly *Corynebacterium segmentosum* (48% relative abundance), and cluster VI was predominantly *Corynebacterium propinquum* (42% relative abundance). Multinomial logistic regression indicated that all community state clusters were associated with woman-child status, meaning there are no differences in the community states associated with HIV status, time point, infection, or antibiotic use. Specifically, clusters I, III, and IV were majority child samples (88%, 92%, and 77% children, respectively), while clusters II, V, and VI were majority woman samples (90%, 94%, and 59% women, respectively; Fig. 3, see green versus pink in the first row below the abundance plots, model *P*-values ranging from 0.01 to 0.05). None of the community states were associated with HIV infection (row 2), time point (row 3), SARS-CoV-2 infection (row 4), or antibiotic usage, including cotrimoxazole (row 5). Taken altogether, these results indicate that community state is most influenced by woman-child status, and further analyses should be stratified by woman-child sample.

### Diversity and richness of the nasopharyngeal bacterial microbiome in women and children

**Fig 4:**
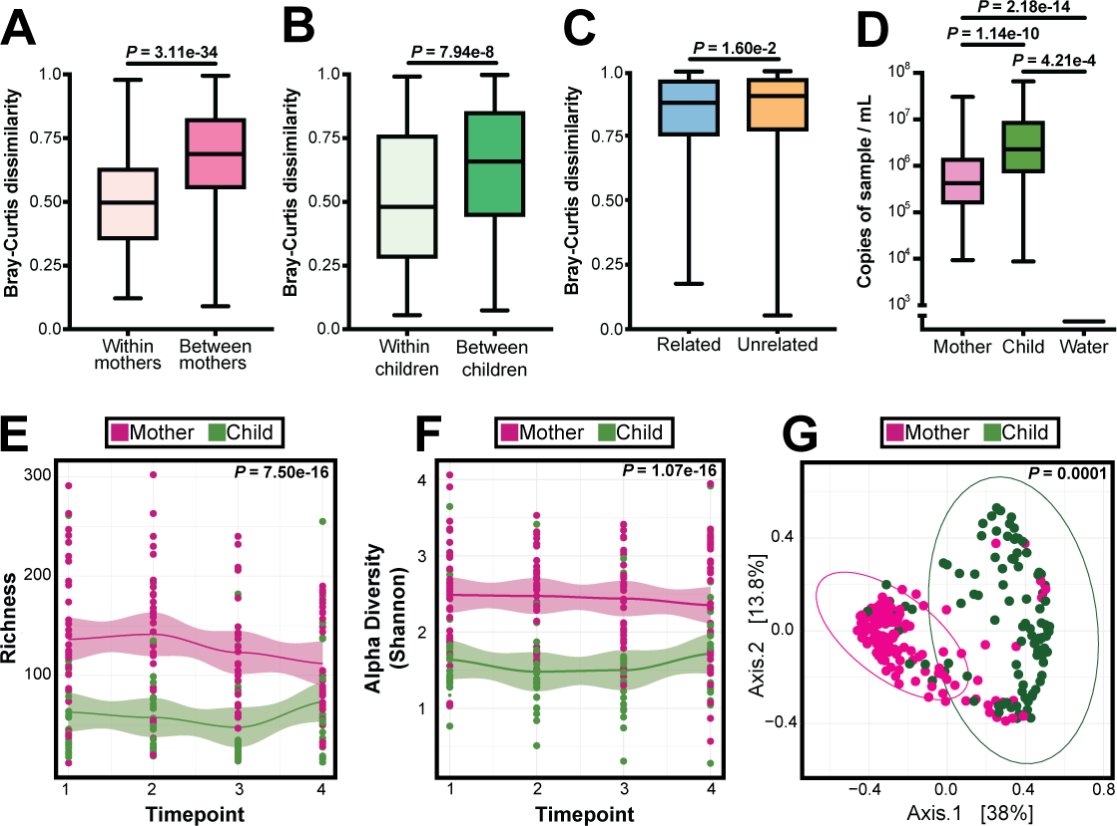
Microbiome analysis of mothers and children. (**A**) Bray-Curtis distance for species within individual mothers and between mothers. Statistical significance assessed by Mann-Whitney test. (**B**) Bray-Curtis distance for species within individual children and between children. Statistical significance assessed by Mann-Whitney test. (**C**) Bray-Curtis distance for species within related child and between unrelated mother-child. Statistical significance assessed by Mann-Whitney test. (**D**) 16S rRNA gene quantitative PCR. 16S rRNA gene copies per sample were quantified in the nasopharyngeal swab samples and water (negative control) samples. Statistical significance was assessed by the Mann-Whitney test, corrected for multiple comparisons using Benjamini-Hochberg. (**E**) Loess plot of bacterial richness against time points in mothers and children. Statistical significance assessed by linear mixed model. (**F**) Loess plot of alpha diversity against time points in mothers and children. Statistical significance assessed by linear mixed model. (**G**) PCoA comparing mother and child samples using weighted Bray-Curtis distance. Statistical significance was assessed by PERMANOVA.

We next considered within-person and between-person microbiome variation between women, children, and family dyads using all samples from all individuals. Beta diversity (weighted Bray-Curtis dissimilarity) was greater when comparing samples between different women than when comparing all samples from an individual woman (*P*-value = 3.11e−34; Fig. 4A). Similarly, beta diversity was significantly greater when comparing samples between different children than when comparing all samples from an individual child (*P*-value = 7.94e−8; Fig. 4B). In addition, when comparing women and children using Bray-Curtis dissimilarity, we observed high dissimilarity between women and children; however, beta diversity was more similar within mother-child dyads than between dyads (*P*-value = 1.6e−2; Fig. 4C). Additionally, principal coordinates analysis (PCoA) of weighted Bray-Curtis dissimilarity showed clearly distinct clustering of women’s samples apart from children’s samples (permutational multivariate analysis of variance [PERMANOVA], *P*-value = 0.001; Fig. 4G). Together, these results show that children and women have lower within-person and within-family variation than between-person variation.

We then considered the differences in bacterial biomass (assayed by 16S quantitative polymerase chain reaction [qPCR]), richness (measured by number of observed bacterial species), and Shannon diversity (measured by Shannon diversity index) between children and women. Children had significantly higher bacterial biomass than women (*P* = 1.39e−10; Fig. 4D). However, women had significantly higher richness (*P*-value = 7.50e−16) and Shannon diversity index than children (*P*-value = 1.07e−16), which were consistent over time (Fig. 4E and F). Taken together, these data suggest the child nasopharyngeal microbiome is more densely populated with fewer bacterial species, whereas the microbiome of adult women is richer and more diverse with less overall biomass, and that these differences were stable over time.

### Impact of HIV on nasopharyngeal microbiome

**Fig 5:**
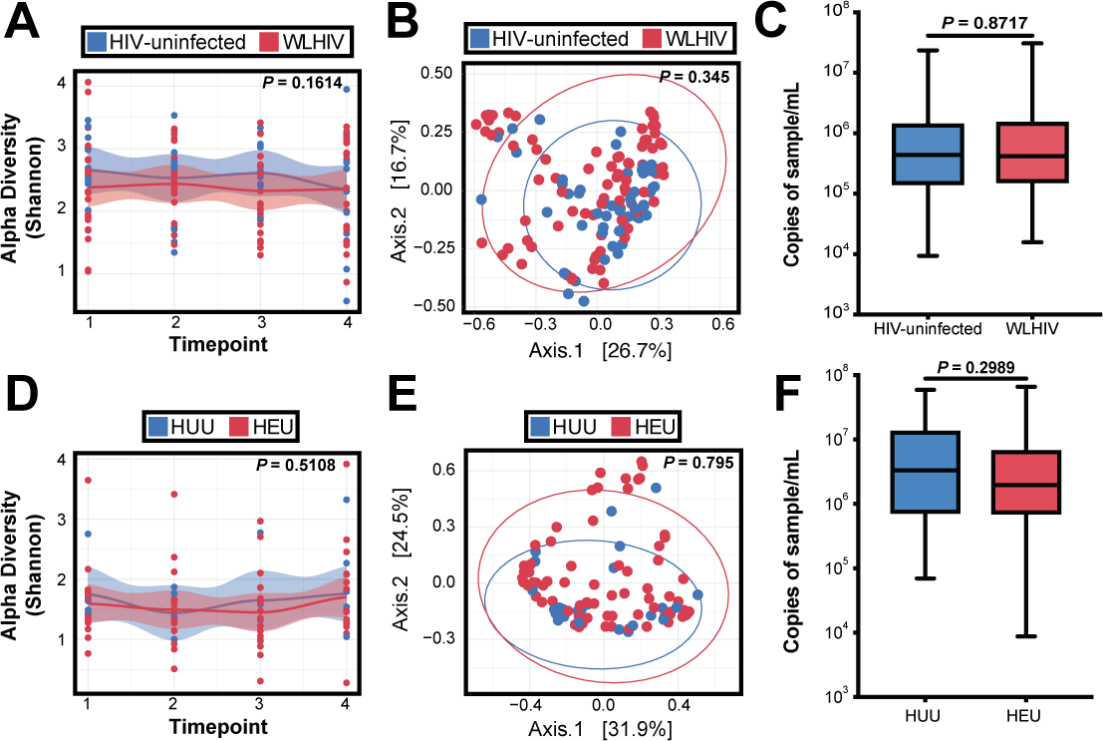
HIV infection and stability in the nasopharyngeal microbiome. (**A**) Loess plot of alpha diversity against time points in women color-coded by HIV status. Statistical significance assessed by linear mixed model. (**B**) PCoA comparing WLHIV/HIV-negative women samples using weighted Bray-Curtis distance. Statistical significance was assessed by PERMANOVA. (**C**) 16S rRNA gene qPCR bacterial load in WLHIV/HIV-negative women. Statistical significance was assessed by the Mann-Whitney test. (**D**) Loess plot of alpha diversity against time points in infants color-coded by HIV status. Statistical significance assessed by linear mixed model. (**E**) PCoA comparing HEU/HUU samples using weighted Bray-Curtis distance. Statistical significance was assessed by PERMANOVA. (**F**) 16S rRNA gene qPCR bacterial load in HEU/HUU. Statistical significance was assessed by Mann-Whitney test.

We next sought to determine whether HIV infection (women) or HIV exposure (children) has an impact on the nasopharyngeal microbiome of women and children using all samples from all participants. HIV-uninfected women consistently had a higher Shannon diversity index than WLHIV. However, the difference was small and not statistically significant (*P*-value = 0.1614; Fig. 5A). PCoA of weighted Bray-Curtis dissimilarity (Fig. 5B) did not show clustering based on HIV status (PERMANOVA, *P*-value = 0.345) in women. Additionally, there was no difference in bacterial biomass between WLHIV and HIV-uninfected women (*P*-value = 0.8717; Fig. 5C). Furthermore, we performed differential abundance testing at the species level between WLHIV and HIV-uninfected women, as well as HEU/HUU children, and there were no reported significant differences. Additionally, for WLHIV, we incorporated CD4 count at enrollment and antiretroviral therapy (ART) regimen, and there were no reported significant associations with either CD4 count or ART. Similarly, in children, we saw no change in Shannon diversity index (*P*-value = 0.5108) between HEU children and HUU children (Fig. 5D), and there was no clustering by HIV exposure status in the PCoA (PERMANOVA, *P*-value = 0.795; Fig. 5E). There was also no difference in bacterial biomass between HEU children and HUU children (*P*-value = 0.2989; Fig. 5F). Together, these results suggest living with, or exposure to, maternal HIV infection treated with optimized ART does not have an impact on the taxonomic characteristics of the nasopharyngeal microbiome among women or children.

### Longitudinal dynamics of the nasopharyngeal microbiome and the impact of SARS-CoV-2

We then examined whether temporal changes in the linear trajectories (slopes) of the Shannon diversity index and richness (number of observed microbial species) of the nasopharyngeal microbiome were associated with incident SARS-CoV-2 infection, HIV exposure, or antibiotic use over the 6-month period the samples were collected. Analyses were conducted separately for women and children. Among women, neither richness nor Shannon diversity index changed significantly over time, and there was no association between SARS-CoV-2 infection or antibiotic usage (*P*-values ranging from 0.0897 to 0.9277). There was a significant association between richness and HIV status in WLHIV (*P* = 0.0494), but the relationship was not significant in Shannon diversity. In children, there was also no significant change over time in richness or Shannon diversity index, and neither SARS-CoV-2 infection nor HIV exposure was associated with change in Shannon diversity index or richness (*P*-values ranging from 0.1002 to 0.8819). We also looked at the linear mixed-effects (LME) model incorporating child age, in months. There was an association between age and richness (*P* = 0.0218), but this significance was lost when analyzing diversity (*P* = 0.2759). Convergence of the child nasopharyngeal microbiome to that of adult women is expected, but the period in which the children were sampled was too short to detect the child microbiome reaching adult maturity. There was an association between the Shannon diversity index and those who were using antibiotics (*P* = 0.0289), but this significance was not seen in bacterial richness.

Additionally, we examined temporal changes in Bray-Curtis dissimilarity of the nasopharyngeal microbiome associated with incident SARS-CoV-2 infection, HIV exposure, or antibiotic use over the 6-month period the samples were collected. Analyses were also conducted separately for women and children. Among women, there was no significant change in Bray-Curtis dissimilarity, and there was no association between SARS-CoV-2 infection, HIV infection, or antibiotic exposure (*P*-values ranging from 0.095 to 0.345). In children, there was also no significant change in Bray-Curtis dissimilarity, and there was no association between SARS-CoV-2 infection, HIV exposure, or antibiotics exposure (*P*-values ranging from 0.338 to 0.795). We also ran the model adding in age of child as their nasopharyngeal microbiome has not yet reached full maturity, though there was no significant association (*P* = 0.350). Together, these data suggest the child and maternal nasopharyngeal microbiomes at the taxonomic bacterial level were stable throughout the period of observation and were not influenced by SARS-CoV-2 infection, maternal HIV status, or child HIV exposure or antibiotic use.

While the overall dynamics were not impacted by SARS-CoV-2, we next compared the Shannon diversity index in pre-infection time points to the SARS-CoV-2 positive time points to assess transient changes in the nasopharyngeal microbiome. We also compared the nasopharyngeal microbiome before, during, and after SARS-CoV-2 infection between SARS-CoV-2-positive individuals to controls who never acquired SARS-CoV-2 infection. To do so, we constructed a longitudinal sampling timeline using the nasopharyngeal swabs, with two time points from the 2 weeks pre-infection, one time point during documented SARS-CoV-2 infection, and one time point an average of 38 days post-infection (Fig. S2A). All individuals positive for SARS-CoV-2 had no reported respiratory symptoms (“symptomatic timepoints”) at the time of infection. For the controls, the timing was similar, but we substituted the SARS-CoV-2 infection sample with either a time point at which the mother or child had reported respiratory symptoms (Fig. S2B) or a matched calendar time point if there were no reported respiratory symptoms (Fig. S2C).

**Fig 6:**
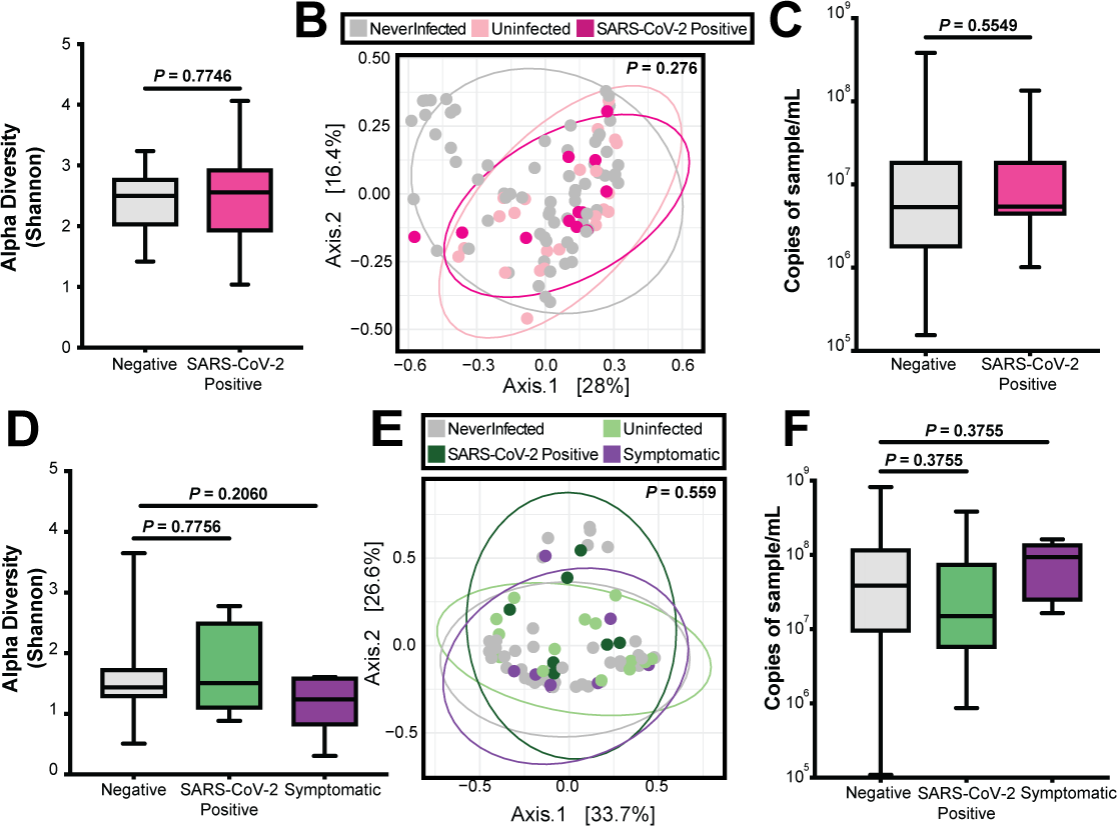
SARS-CoV-2 impact on the nasopharyngeal microbiome. (**A**) Alpha diversity in women comparing all negative samples to the first positive SARS-CoV-2 sample. Statistical significance was assessed by the Mann-Whitney test. (**B**) PCoA in women comparing all never-positive, negative, and SARS-CoV-2-positive samples. Statistical significance was assessed by PERMANOVA. (**C**) Bacterial 16S rRNA gene qPCR bacterial load in women comparing all negative samples and SARS-CoV-2-positive samples. Statistical significance was assessed by the Mann-Whitney test. (**D**) Alpha diversity in children comparing all negative samples to the first positive SARS-CoV-2 sample and first symptomatic sample. Statistical significance was assessed by the Mann-Whitney test, correcting for multiple comparisons using Benjamini-Hochberg. (**E**) PCoA in children comparing all never-positive, negative, SARS-CoV-2-positive, and symptomatic samples. Statistical significance was assessed by PERMANOVA. (**F**) 16S rRNA gene qPCR bacterial load in children comparing all negative samples, SARS-CoV-2-positive samples, and symptomatic samples. Statistical significance was assessed by the Mann-Whitney test correcting for multiple comparisons using Benjamini-Hochberg.

In women, there was no significant difference in the Shannon diversity index (*P*-value = 0.7746) when comparing pre-infection time points to the time point of SARS-CoV-2 infection (Fig. 6A). These results were also supported by PCoA of weighted Bray-Curtis dissimilarity, which did not show clustering of negative samples from never-positive women, negative samples from women with SARS-CoV-2 infection, or SARS-CoV-2-positive samples (PERMANOVA, *P*-value = 0.276; Fig. 6B). There was also no significant difference (*P*-value = 0.5549) in bacterial load when comparing women’s negative samples to positive SARS-CoV-2 samples (Fig. 6C). Similarly, there was no statistically significant difference in the Shannon diversity index among children when comparing negative time points to SARS-CoV-2-infected time points (*P*-value = 0.7756), nor was there a difference between negative time points and symptomatic time points (*P*-value = 0.2060) (Fig. 6D). There was no clustering of negative samples from never-positive children, negative samples from children with SARS-CoV-2 infection, SARS-CoV-2-positive samples, or samples from time points with reported respiratory symptoms (PERMANOVA, *P*-value = 0.559; Fig. 6E). Lastly, we compared bacterial load at pre-infection time points, SARS-CoV-2 time points, and symptomatic time points (Benjamini-Hochberg-corrected *P*-values = 0.3755) in children (Fig. 6F). Taken together, we conclude that neither SARS-CoV-2 infection nor undiagnosed respiratory infections caused dysbiosis of the nasopharyngeal bacterial microbiome from both negative samples and never-positive samples.

**Fig 7:**
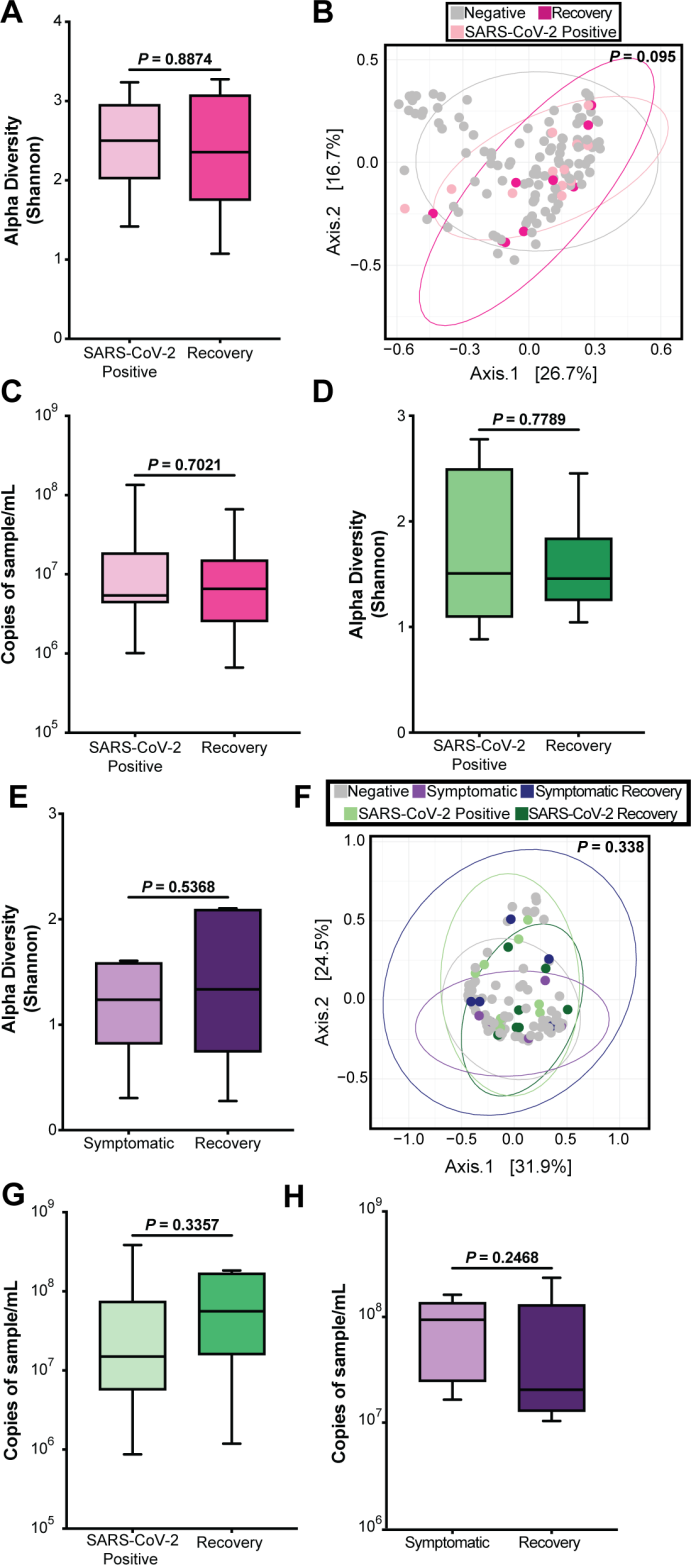
Recovery of the nasopharyngeal microbiome post-infection. (**A**) Alpha diversity in women comparing the first positive SARS-CoV-2 sample to the first negative sample post-infection. Statistical significance was assessed by the Mann-Whitney test. (**B**) PCoA in women comparing negative, SARS-CoV-2-positive, and recovery samples. Statistical significance was assessed by PERMANOVA. (**C**) Bacterial 16S rRNA gene qPCR bacterial load in women comparing SARS-CoV-2-positive samples and recovery samples. Statistical significance was assessed by the Mann-Whitney test. (**D**) Alpha diversity in children comparing the first positive SARS-CoV-2 sample to the first negative sample post-infection. Statistical significance was assessed by the Mann-Whitney test. (**E**) Alpha diversity in children comparing the first symptomatic sample to the first non-symptomatic sample. Statistical significance was assessed by the Mann-Whitney test. (**F**) PCoA in children comparing negative, SARS-CoV-2-positive, symptomatic, and recovery samples. Statistical significance was assessed by PERMANOVA. (**G**) 16S rRNA gene qPCR bacterial load in children comparing first positive SARS-CoV-2 sample to recovery. Statistical significance was assessed by the Mann-Whitney test. (**H**) 16S rRNA gene qPCR bacterial load in children comparing symptomatic samples to recovery. Statistical significance was assessed by the Mann-Whitney test.

Though we did not detect a change in the nasopharyngeal microbiome at the time of SARS-CoV-2 infection, SARS-CoV-2 can cause long-term sequelae, which led us to ask whether there were effects on the microbiome after infection. The next available subsequent follow-up sample (average 38 days post-infection) was termed the “recovery” time point, and all recovery time points were negative for SARS-CoV-2 RNA. The Shannon diversity index of the nasopharyngeal microbiome remained stable in women even after SARS-CoV-2 recovery (*P*-value = 0.8874; Fig. 7A). PCoA on weighted Bray-Curtis dissimilarity showed no discernible clustering (PERMANOVA, *P* = 0.095) in women when comparing negative SARS-CoV-2 samples and recovery samples (Fig. 7B). There was also no statistical difference (*P*-value = 0.7021) between SARS-CoV-2-infected time points and recovery time points when looking at bacterial load (Fig. 7E). Neither children infected with SARS-CoV-2 (*P*-value = 0.7789‘ Fig. 7D) nor children who were symptomatic for respiratory illness showed a difference in Shannon diversity index (*P*-value = 0.5368) from the time of infection to post-infection (Fig. 7E). No clustering was apparent with PCoA on weighted Bray-Curtis dissimilarity between negative SARS-CoV-2 samples and symptomatic children and their respective recoveries (Fig. 7F). Finally, we looked at the differences in bacterial load between infected samples and all available recovery samples. There was no statistical difference in children between both SARS-CoV-2-infected time points and symptomatic time points and their respective recoveries (*P*-values = 0.3357 and 0.2468, respectively; Fig. 7G and H). Taken together, these results show that SARS-CoV-2 infection does not have a lasting impact on the taxonomic characteristics of the nasopharyngeal microbiome and suggest the nasopharyngeal microbiome is resilient in both mothers and children, maintaining stability before, during, and after SARS-CoV-2 infection.

### Specific bacterial species that differentiate the nasopharyngeal microbiome in women and children

**Fig 8:**
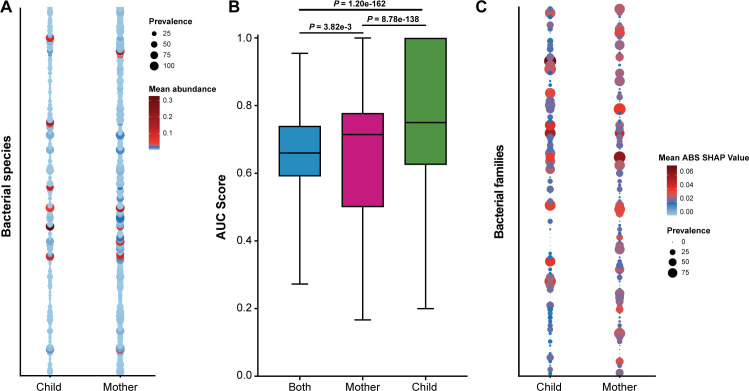
Discriminant analysis on the nasopharyngeal microbiome. (**A**) Prevalence and abundance plot of statistically significant bacterial species determined by MaAsLin2. Size of circle represents prevalence, and color represents bacterial species abundance. (**B**) Boxplot showing mean area under the curve (AUC) scores. (**C**) Prevalence and abundance plot of statistically different families according to SARS-CoV-2 infection status determined by machine learning (ML) methods. The size of the circle represents prevalence, and color represents bacterial family taxa abundance.

Lastly, we performed a multivariate analysis using MaAsLin2 and used novel machine-learning methods to identify discriminating taxa among women and children and by SARS-CoV-2 infection status. Additionally, there were no discriminant taxa associated with time point, infection, HIV status, or antibiotic usage. We identified 197 statistically significant discriminating bacterial species (Table S3) that differed based on mother-child status (Fig. 8A). Of these, *Moraxella nonliquefaciens, Moraxella catarrhalis, Haemophilus influenzae, Streptococcus pneumoniae,* and *Dolosigranulum pigrum* were more common in children, while *Staphylococcus epidermidis, Staphylococcus aureus, Cutibacterium acnes, Corynebacterium segmentosum*, and *Corynebacterium macginleyi* were more common in mothers.

We also used novel machine-learning methods to identify discriminating taxa associated with SARS-CoV-2 infection. Area under the curve (AUC) scores of the best models for combined and stratified data sets confirmed the models should be stratified between women and children (Fig. 8B). We identified 84 taxa that differed at the familial level according to SARS-CoV-2 infection status (Table S4) and plotted the mean Shapley additive explanations (SHAP) values for both women and children (Fig. 8C). Together, these data corroborate our previous finding that the microbiome composition of women is different from that of children and suggest that SARS-CoV-2 may impact the nasopharyngeal microbiome through smaller compositional changes at the familial taxa level rather than larger changes typically captured by changes in Shannon diversity index, beta diversity, or community states.

## DISCUSSION

In this study, we investigated the impact of SARS-CoV-2 on the nasopharyngeal bacterial microbiome in Kenyan WLHIV, HIV-uninfected women, and their HEU and HUU children. The nasopharyngeal microbiome was resilient throughout SARS-CoV-2 infection in both mothers and children, though mothers and children differed in microbiome composition, diversity, and richness.

We first aimed to elucidate the genomic epidemiology of SARS-CoV-2 within our cohort and then comparatively to Kenya as a whole. Fifteen individuals in the cohort were infected with SARS-CoV-2 Omicron, and one individual was infected with SARS-CoV-2 Delta (Fig. 2A). Of note, we found that only three mother-child dyads were positive with the same strain of SARS-CoV-2, indicating the possibility of within-household transmission; the other 14 dyads had discordant transmission, demonstrating within-household transmission is not inevitable. Proportions of concordant and discordant infections among dyads in this cohort, measured via SARS-CoV-2 serology, have been reported previously and showed maternal infection was associated with roughly a twofold increased risk of infant infection (29). Most documented infections in our study cohort were within the same week in December, coinciding with the month Kenya reported the most SARS-CoV-2 cases and deaths (13). By analyzing the transmission networks, these infections were estimated to coincide with the rapid spread of Omicron across the country, rather than geographic location (12, 13) (Fig. 2C and D).

The nasopharynx harbors a bacterial microbiome with core species shared across individuals that are vital to developing a functional respiratory system that is resilient to disease (5), though we observed that there are distinct differences in microbial composition between adults and children (Fig. 3). The infant nasopharyngeal microbiome is predominated by *Dolosigranulum pigrum, Moraxella nonliquefaciens,* and *Haemophilus influenzae* (31). *Dolosigranulum pigrum* is a commonly found commensal bacterium in the developing nasopharyngeal microbiome. *Moraxella nonliquefaciens* is common in the developing nasopharynx, though there is conflicting research on whether or not it is a commensal organism or indicative of future dysbiosis or infections (32). While *Haemophilus influenzae* is considered pathogenic, it commonly colonizes the developing nasopharyngeal microbiome (9). In our study, the adult woman nasopharyngeal microbiome was predominated by *Staphylococcus epidermidis, Corynebacterium segmentosum,* and *Corynebacterium propinquum*. Studies have shown species belonging to the *Staphylococcus* and *Corynebacterium* genera are common in the mature nasopharyngeal microbiome (33).

Additionally, we found that the nasopharyngeal microbiome differed between women and children in richness, diversity, and bacterial load, and that there was less within-person than between-person variation within mothers, children, and families (Fig. 4A through C). There is no consensus as to when the nasopharyngeal microbiome reaches maturity, the range being anywhere from early childhood to late adolescence (34, 35). Here, we are only able to assess the dynamics of nasopharyngeal microbiome maturity during a few months in children aged 17–25 months. Most studies aiming to characterize the infant microbiome do so with the intent of identifying dysbiosis that may represent further illness, with much of the data having no clear consensus on what bacteria are pathogenic or commensal (32). As stated previously, the cohort, excluding the six children who were symptomatic, was largely asymptomatic, including those who were infected with SARS CoV-2. Our study is unique in that we sampled and characterized the stability of the still-developing nasopharyngeal microbiome of healthy children.

We did not find an impact on the nasopharyngeal microbiome from HIV infection among women receiving antiretroviral treatment or HEU children (Fig. 5). Studies have reported a decrease in relative abundance of certain oral microbiota in those who are living with HIV but not receiving ART; however, the difference was diminished when comparing HIV-uninfected individuals to those receiving ART (36), similar to the comparison groups for our study in which all women living with HIV were receiving long-term, optimized ART regimens. In contrast, studies of WLHIV have reported that antibiotics, but not antiretrovirals, have an impact on breast milk microbiome (37). For those living with HIV today, HIV infection and immunosuppression are two separate factors considered for overall health. Though we did not have concurrent data to assess the relationship between CD4 count and nasopharyngeal microbiome composition, our data suggest ART-treated HIV infection does not substantially impact the nasopharyngeal microbiome in the presence or absence of SARS-CoV-2 infection. CD4 counts for this particular cohort have been published previously and were all within the normal range for WLHIV (29).

Multiple studies have looked at the relationship between SARS-CoV-2 infection and the nasopharyngeal microbiome; however, few studies have analyzed this relationship longitudinally. It is important to note that differences in the nasopharyngeal microbiome may vary by geographical location or demographics (25), and no studies to date have focused on the interplay between SARS-CoV-2 and the nasopharyngeal microbiome in Kenyan or African populations. Some studies have suggested that variations in the nasopharyngeal microbiome may be dependent on demographic differences (25). While a few studies (19, 20) found a decrease in alpha and beta diversity in those infected with SARS-CoV-2, others have found the converse to be true (23). Most studies have found no difference in alpha or beta diversity metrics by SARS-CoV-2 infection status but have noticed dysbiosis in the nasopharyngeal microbiome at the bacterial taxonomic level (38, 39). Several studies have found an association between SARS-CoV-2 severity and microbiome dysbiosis (24, 25, 40). The cohort we studied reported little to no symptoms throughout the study (29), which is a plausible reason why we observed stability in the nasopharyngeal microbiome. Symptoms are often correlated with increased inflammation and cytokine storms that can potentially cause further dysbiosis to the microbiome (41).

The strengths of our study include the robust longitudinal and cross-sectional sampling of individuals both living with HIV and those HIV-uninfected, as well as individuals infected with SARS-CoV-2 and those who were healthy throughout the duration of the study. To date, there have been no studies focusing on the interplay of SARS-CoV-2 and HIV in the nasopharyngeal microbiome. This is an important area of study given HIV infection may result in more severe SARS-CoV-2 infection (42). Furthermore, being able to study this interaction while being able to compare those who are living with HIV and did not contract SARS-CoV-2, those who are HIV-uninfected and contracted SARS-CoV-2, and those who are HIV-uninfected and never contracted SARS-CoV-2 allows us to investigate all potential viral interactions and their impact on the nasopharyngeal microbiome. However, there is a significant window of variance from the point of infection to recovery (1 month to 4 months post-infection), which could limit our ability to see any differences in the microbiome. Additionally, our study was able to assess the severity of SARS-CoV-2 infection via reported symptoms at the time of infection. As stated previously, there is a relationship between disease severity, as monitored by symptoms, and nasopharyngeal microbiome disruption (24). Our study is unique in that we were able to study a group of individuals who were non-symptomatic not only at the point of infection but throughout the study period. Conversely, this could also be viewed as a limitation in our analysis as we were not able to directly compare non-symptomatic COVID-19 to symptomatic COVID-19. While we were able to identify four individuals infected with a respiratory virus that was not SARS-CoV-2, we were unable to identify an infectious agent for the other two individuals. This is likely due to the difficulty of identifying viral pathogens via sequencing, and should be considered an additional limitation. Limitations in our study also include the unavailability of certain HIV-specific variables, namely viral load and adult woman cotrimoxazole exposure. Though we did not have systematically collected HIV viral load data, most mothers had normal CD4 counts at enrollment, which suggests well-treated HIV. The adult women in our cohort were also in a transitional period from one ART regimen to another, which could have had impacts on the nasopharyngeal microbiome we could not directly account for. Additionally, another limitation in our study is that we were unable to elucidate the exact window of antibiotic exposure for our study. Depending on whether the weekly visit was recorded as a wellness or COVID-19 visit, individuals were not asked at COVID-19 visits if they were on antibiotics. Additionally, WLHIV and HEU children are often prescribed cotrimoxazole as a prophylactic during periods of high risk, resulting in the majority of HEU children being those who were recorded using antibiotics (30). As stated previously, antibiotics and exposure to them have the potential to impact the nasopharyngeal microbiome, particularly in children (5). Therefore, if we were able to most accurately determine when individuals were taking antibiotics, we would be able to control for any confounding as a result. Given the age of the children in the cohort, most are either not breastfeeding anymore or are mixed with feeding practices; therefore, we are unable to analyze breastfeeding and its impact on the nasopharyngeal microbiome directly. Additionally, while we were able to match our control cohort for HIV status and calendar time, we were unable to match for age for both women and children. Furthermore, given COVID-19 restrictions at the time, not all individuals had trained study staff collect nasal swab samples for all study visits. Another major limitation in our study, despite the robust longitudinal sampling, is the small sample size of infected individuals with SARS-CoV-2 (*n* = 20). With a small sample size, presumably the effects we would be able to observe would be those of a large degree, and any smaller nuances would likely go unnoticed. A larger study cohort with more SARS-CoV-2-positive individuals would make the conclusions we have presented here much stronger.

The nasopharyngeal microbiome is an often-overlooked and under-researched component of the overall human microbiome, garnering increased study interest over the past 3 years since the declaration of COVID-19 as a global pandemic. Dysbiosis in any microbiome, including the nasopharyngeal microbiome, may give rise to opportunistic infections, as well as potential inflammatory responses that may further impact the microbiome. Resilience in the nasopharyngeal microbiome upon respiratory infection from a pathogen, such as SARS-CoV-2, has overall positive implications for human health.

## MATERIALS AND METHODS

### Study population

The Linda Kizazi Cohort was accrued to study how HIV exposure modified establishment of the infant microbiome in breastfed children in the era of universal ART; details of cohort and sampling have been previously published (29). Briefly, pregnant women were enrolled at Mathare North Health Centre in Nairobi, Kenya, and followed with their infant from the third trimester to 24 months postpartum. The adult women in the cohort had well-managed HIV as inclusion criteria for the study included adherence to ART for ≥6 months. Most women in the cohort were receiving the same regimen of TDF + 3TC + DTG; however, the cohort was sampled during a change in the general Kenyan guidelines, and therefore some women were transitioning from the TDF + 3TC + EFV regimen. Detailed information was collected at each study visit to determine clinical symptoms/illnesses, medications, and feeding modality. Most children were not breastfeeding at the time of the nested study, and those who were breastfeeding were in a mixed modality. In this nested sub-study, a total of 74 women and their children provided longitudinal nasal swabs from September 2021 to December 2021, and then had 3 month follow-up visits throughout March 2022. The median age of the women in the cohort was 32, and the median age of the children in the cohort was 21 months (Table S1). All samples collected in this time were tested for SARS-CoV-2 RNA (see below). Samples were selected for microbiome analysis to describe changes during incident SARS-CoV-2 infection: two samples were chosen pre-SARS-CoV-2 infection to establish a microbiome baseline (2 weeks prior to infection), one sample that tested positive for SARS-CoV-2 RNA, and one sample was chosen post-infection to assess impact on the microbiome over time. The samples were chosen over a 6 month period, with most samples being collected from late November to December.

### Nasal swab collection

Mothers self-collected mid-turbinate nasal swab samples from themselves and their children. At each visit, participants received sample collection kits containing a viscose-tip swab with plastic handle and 3 mL of viral transport medium (VTM; Citoswab Collection and Transport Kit, Citoswab, Nanjing, China). Depending on COVID-19 restrictions at the time of sample collection, participants were either able to have their COVID-19 visit at the clinic or in their home, though samples were self-collected regardless of location. Written and picture instructions for proper sample collection were included with the kit, and study clinicians provided verbal guidance by phone for the initial sample and as needed thereafter. Participants were instructed to insert the swab tip into one nostril until they felt slight resistance, rotate the swab in a circle inside the nose for about 15 s, and then immediately place the swab into the vial of VTM, breaking off the swab handle and sealing the cap. Participants were asked to inform study staff if any VTM spilled or if they touched the swab tip. Samples were stored temporarily at 2-8°C for up to 24 hours, then separated into three aliquots of approximately 1 mL volume of VTM before long-term storage at −80°C.

### SARS-CoV-2 testing

We performed real-time reverse transcription polymerase chain reaction on the eMAG (total nucleic acid [TNA]) extracts using the ThermoFisher TaqPath COVID-19 Fast PCR Combo kit 2.0 assay (Waltham, MA) following the manufacturer’s guidelines. The median SARS-CoV-2 viral load for the three genes tested was 25.4 (IQR 22.4–31.9 *N* gene), 26.7 (IQR 24.1–32.1 *ORF1a* gene), and 26.6 (IQR 23.9–30.9 *ORF1b* gene), respectively.

### SARS-CoV-2 sequencing

SARS-CoV-2 sequencing was performed as previously described (12). Next-generation sequencing (NGS) library preparation for samples was performed using the COVIDSeq Test (Illumina, San Diego, CA, USA) with ARTIC v.4 and ARTIC v.4.1 primers (43). Libraries were sequenced on the Illumina NextSeq 2000 instrument using 2 × 109 paired-end reads. Adapter sequences were trimmed using Trim Galore version 0.6.5 (44), aligned to the Wuhan1 reference genome using the Burrows-Wheeler Aligner, BWA-MEM version 0.7.17-r1188 (45), and had their primer sequences trimmed using iVAR version 1.3.1 (46). Consensus sequences were generated using SAMtools (47) and iVAR software packages. Lineage determinations were performed with pangolin software (48). Sequence quality was validated and annotated using VADR version 1.4 (49).

### Respiratory virus sequencing

Samples negative for SARS-CoV-2 but collected at time points with any reported symptoms of respiratory infection, of which we have highlighted fever, cough, and/or shortness of breath, were enriched for respiratory virus nucleic acids using the Illumina Respiratory Virus Oligo Panel v.2 (San Diego, CA). Libraries were sequenced on an Illumina NextSeq 2000 instrument using 2 × 109 paired-end reads. Sequences were trimmed of adapter sequences, and host reads were removed using bbtools (50). Reads were aligned to panel genomes using bowtie2 version 2.2.5 (51). Consensus sequences were generated using SAMtools (47) and iVAR (46) software packages. Samples with over 1,000 counts and genome-wide coverage were considered positive for infection.

### Microbiome extraction and sequencing

A total of 750 µL of NP swab in viral transport medium was transferred into a Qiagen PowerBead Tube and centrifuged at 13,000 *× g* for 5 minutes at 4°. A total of 500 µL of supernatant was transferred into a bioMérieux eMAG (Marcy-l'Étoile, France) for TNA extraction. DNA extraction was performed on the NP swab pellet using the DNeasy PowerSoil kit (Qiagen). Nucleic acid extracts were stored at −80°C then thawed on ice at the time of analysis. Negative controls (phosphate-buffered saline [PBS]) were processed in parallel to the nasal swab samples to assess contamination during extraction, amplification, and sequencing. All samples were sequenced using whole-genome metagenomic sequencing. Libraries were sequenced on the Illumina NextSeq2000 instrument using 2 × 150 paired-end reads. Human reads were removed using Bowtie2 version 2.2.5 (51) and SAMtools (47).

### Phylogenetic and network analysis of SARS-CoV-2 sequences

To generate the phylogenetic tree, SARS-CoV-2 genome sequences were first aligned using MAFFT software version 7.520 (52) using default arguments. Phylogenetic analysis was performed with IQTREE 2.2.2.3 (53), using default arguments and ModelFinder algorithm (54), and visualized using FigTree v.1.4.4 (55). The sole Delta lineage sequence was designated as the outgroup. Viral transmission networks were generated as previously reported (12). A total of 2,336 GISAID submissions for SARS-CoV-2 were downloaded on 20 June 2022 using text and collection parameters. Text: “Africa/Kenya.” Collection: 2021-29-November to 2021-31-December, and “Complete” genome coverage. Briefly, genome sequences were aligned using MAFFT software version 7.520, using default arguments. A maximum-likelihood distance matrix was created using IQTREE2 version 2.2.2.3, with the -m TIM+F+R argument. The distance matrix was converted to an edge list, and source/target pairs exceeding a genetic distance of 6E-05 or having collection dates differing by over 7 days were removed. The network layout was arranged in Cytoscape (v.3.10.0) (56) using a perfuse force-directed layout with genetic distance as edge weights.

### Bacterial microbiome analysis

A median of 7,058,076 (IQR = 2,408,619–26,567,952) reads were quality filtered using BBtools and Cutadapt (57, 58). These reads were then input to KrakenUniq (59) using a custom database (RefSeq archaeal, bacterial, viral, plasmid, fungal genomes, and the GRCh38 human genome, downloaded January 2022) to output read counts for assigned bacterial taxa. We then used DBSCAN, a clustering algorithm, to obtain the cutoff for optimal number of unique k-mers. For our data, anything below 1,800 k-mers per taxon was removed and parsed the data at the species level. We then used the R package decontam as previously described (1.20.0) to remove the default called contaminants (60).

### Linear mixed-effects and PERMANOVA models

To account for repeated sampling, we used LME models (R package nlme version 3.1.149) to compare changes in richness and alpha diversity over time (time point) for both women and children. We used PERMANOVA, accounting for repeated measures (vegan, permute, and Adonis in R) to assess changes in beta diversity as measured by weighted Bray-Curtis dissimilarity. Family member (woman or child), HIV status, antibiotic use, ever/never SARS-CoV-2 infection, and timepoint-specific SARS-CoV-2 infection status were variables used in all models to account for exposure or possible confounding. For the models, two different methods were used to classify SARS-CoV-2 samples: SARS-CoV-2 infection status at sample collection time and if individuals were ever/never infected with SARS-CoV-2. We first performed LME and Adonis models with family member status to see if there was a significant difference between women and children and stratified for subsequent analyses. Linear and PCoA plots were plotted using ggplot2 (version 3.4.3), and boxplots were made using GraphPad Prism version 10.0.2.

### Community state analysis

To obtain the community states present in nasal swab samples of this study, we clustered our data with the k-means method. We used the stats function k-means to both calculate the sum of squares to determine the optimal number of clusters as well as cluster the relative abundance at family level into six groups. To determine associations between community states and family member, HIV status, time point in study, infection status, and antibiotic use, we used the R package mclogit (version 0.8.7.2) to perform multinomial logit models with random effects for patient IDs. Age was not used in the model as it was highly correlated with family member (mother or child status). The Benjamini-Hochberg method was used to correct for multiple comparisons. The relative abundance graph was made using GraphPad Prism version 10.0.2.

### Differential analysis

To find differentiating bacterial species, we created models using the R package Microbiome Multivariable Association with Linear Models (MaAsLin2, version 1.14.1) with metadata controlling for longitudinal samples per patient. Person code, time point, infection status, ever/never infected, HIV status, and antibiotic usage were additional variables included in the model. We used the default q-value threshold of 0.25 for significance, then stratified by between women and child status. We recalculated the q-value using all the results output file and used the updated q-value of 0.05 for significance per factor. Differential abundance plots were plotted using ggplot2 (version 3.4.3).

### qPCR assay

SYBR Green quantitative PCR for the 16S rRNA gene was performed using primers 515F (5′-GTGYCAGCMGCCGCGGTAA-3′) and 806R (5′-GGACTACNVGGGTWTCTAAT-3′). The 15 µL reaction included 5 µL of extracted DNA and 0.4 µL (100 µm) of each primer, 10 µL of ThermoFisher Fast SYBR Green (Waltham, MA), and 4.2 µL of water. The following cycling conditions were used: 95°C for 20 s, then 40 cycles of 95°C for 1 s, and 60°C for 20 s followed by a melt curve. Samples were tested in two 96-well plates with 12 water-only negative controls and two positive gBlock controls. gBlock nucleic acids were synthesized by IDT (Coralville, IA) using *Verrucomicrobia bacterium* 16S V4 gene sequence (*n* = 292). The positive threshold value (<40) was determined from gBlock serial dilutions of 10^8^–10^1^ genome copies per microliter. All water controls were below the limit of detection. qPCR boxplots and corresponding Mann-Whitney tests were done using GraphPad Prism version 10.0.2.

### Machine learning

Machine learning analyses were performed on the Sol supercomputer at Arizona State University (ASU) (61). Normalized reads parsed to the familial taxa were used as the input data and went through natural log transformation with an arbitrary minimal pseudo count added. The model had the best performance using taxa parsed to the familial level due to the quantity of bacterial families present in the cohort, when compared to the more quantitatively massive genera and species present. The data set was stratified according to woman/child status, and SARS-CoV-2 infection status was used as the predicting label. Only those infected with SARS-CoV-2 were included in the machine-learning model. The first three time points, including time of infection, were used in machine-learning analyses. For each machine-learning model, a fourfold cross-validation was run on each data set 1,000 times independently (62), and multilayer perceptron (MLP) archived the best average AUC. The best model went through SHAP analysis 1,000 times independently to obtain average SHAP scores for important operational taxonomic units (OTUs). Models selected to participate in the GridSearchCV process are Random Forest, AdaBoost, Support Vector Machine, MLP from scikit-learn (v.1.3.2), XGBoost (v.1.7.4), LightGBM (v.4.0.0), CatBoost (v.1.2), and SHAP (v.0.42.1). Differential abundance plots were plotted using ggplot2 (version 3.4.3), and AUC scores were graphed using GraphPad Prism version 10.0.2.

## Data Availability

The STORMS checklist is available at https://github.com/ASU-Lim-Lab/LindaKizazi-NP-COVID-19-HIV. Sequence data have been deposited to the NCBI Sequence Read Archive under accession number PRNJA1078389. SARS-CoV-2 genome sequences have been deposited to the GISAID repository under accession EPI_ISL_18915178 to EPI_ISL_18915193: https://doi.org/10.55876/gis8.250404pz. Code to reproduce analysis and plots is available at https://github.com/ASU-Lim-Lab/LindaKizazi-NP-COVID-19-HIV.

## SUPPLEMENTAL MATERIAL

Figure S1 msystems.01568-24-s0001.tif Comprehensive sampling and SARS-CoV-2 testing schema of complete cohort September 2021-April 2022.

Figure S2 msystems.01568-24-s0002.tif Detailed timeline from September 2021-April 2022.

Legends msystems.01568-24-s0003.docx Supplemental figure legends.

Supplemental Tables msystems.01568-24-s0004.xlsx Tables S1-S4.

ASM does not own the copyrights to Supplemental Material that may be linked to, or accessed through, an article. The authors have granted ASM a non-exclusive, world-wide license to publish the Supplemental Material files. Please contact the corresponding author directly for reuse.
