# Supplementary figures and images for: Longitudinal dynamics of the nasopharyngeal microbiome in response to SARS-CoV-2 Omicron variant and HIV infection in Kenyan women and their children
Source: mSystems. 2025 Apr 22;10(5):e01568-24. doi: 10.1128/msystems.01568-24 (PMC12090742; doi:10.1128/msystems.01568-24)

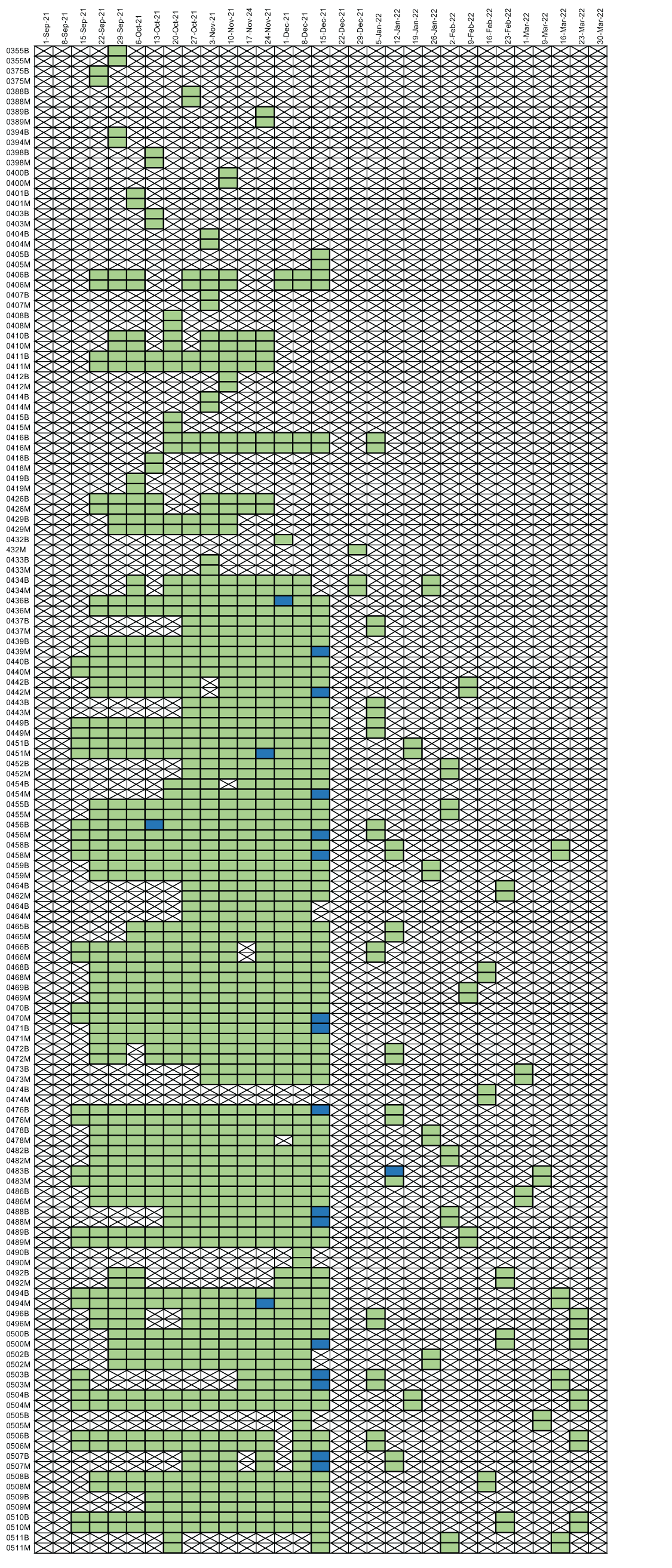

Supplement: Figure S1 — Comprehensive sampling and SARS-CoV-2 testing schema of complete cohort September 2021-April 2022. [file msystems.01568-24-s0001.tif]

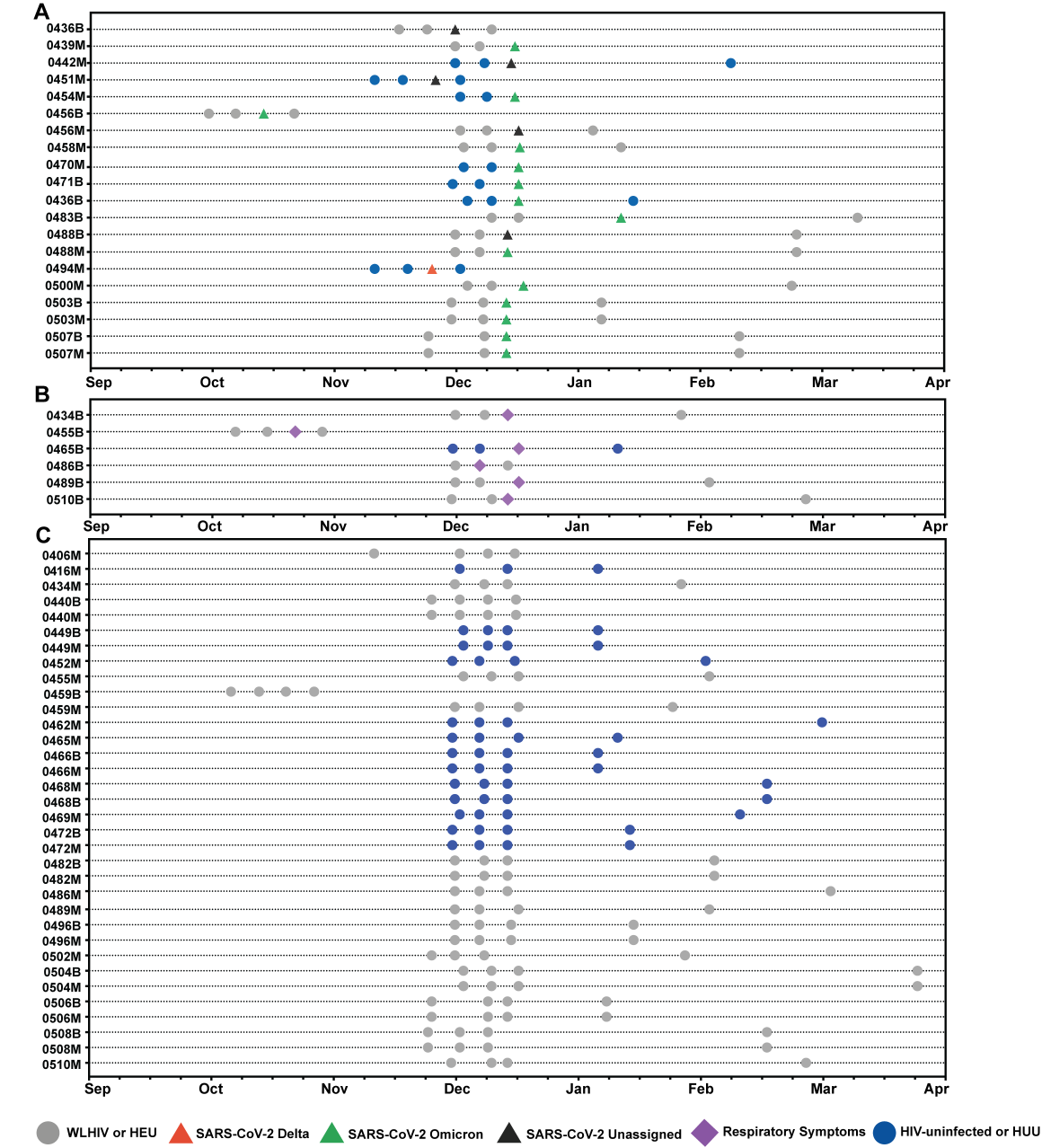

Supplement: Figure S2 — Detailed timeline from September 2021-April 2022. [file msystems.01568-24-s0002.tif]
