# Supplementary material for: Longitudinal dynamics of the nasopharyngeal microbiome in response to SARS-CoV-2 Omicron variant and HIV infection in Kenyan women and their children
Source: mSystems. 2025 Apr 22;10(5):e01568-24. doi: 10.1128/msystems.01568-24 (PMC12090742; doi:10.1128/msystems.01568-24)
Supplement: Legends — Supplemental figure legends. [file msystems.01568-24-s0003.docx]

**Supplementary Figure Legends**

**Supplemental Figure 1:** Comprehensive sampling and SARS-CoV-2 testing schema of complete cohort September 2021- April 2022. Anonymized ID numbers on y-axis for mothers (M) and children (B). Calendar time is shown on the x-axis. Green rectangles denote nasopharyngeal swab specimens that test negative for SARS-CoV-2 by ThermoFisher TaqPath COVID-19 Fast PCR Combo kit 2.0 assay, SARS-CoV-2 positive specimens are denoted in blue, and specimens that were not available are indicated with crossed out rectangles.

**Supplemental Figure 2:** Detailed timeline from September 2021-April 2022. (A) Anonymized individuals who tested positive for SARS-CoV-2. (B) Anonymized individuals who were symptomatic, but SARS-CoV-2 negative. (C) Anonymized individuals who were neither symptomatic nor SARS-CoV-2 positive. Two sequential previous timepoints from point of infection were used to determine microbiome stability, and one sequential timepoint post infection was used for recovery. Anonymized ID numbers on y-axis for mothers (M) and children (B). Blue circles denote either HIV-uninfected mothers or HUU children, grey circles denote WLHIV or HEU children. Red triangles denote infection with SARS-CoV-2 Delta, green triangles denote infection with SARS-CoV-2 Omicron, and black triangles denote an unassigned SARS-CoV-2 infection. Purple diamonds denote a timepoint where the individual was experiencing respiratory symptoms. Calendar time is on the x-axis.
